# Genetic diversity, linkage disequilibrium, and population structure analysis of the tea plant (*Camellia sinensis*) from an origin center, Guizhou plateau, using genome-wide SNPs developed by genotyping-by-sequencing

**DOI:** 10.1186/s12870-019-1917-5

**Published:** 2019-07-23

**Authors:** Suzhen Niu, Qinfei Song, Hisashi Koiwa, Dahe Qiao, Degang Zhao, Zhengwu Chen, Xia Liu, Xiaopeng Wen

**Affiliations:** 1 The Key Laboratory of Plant Resources Conservation and Germplasm Innovationin Mountainous Region (Ministry of Education), Institute of Agro-Bioengineering / College of Tea Science, Guizhou University, Guiyang, 550025 Guizhou Province People’s Republic of China; 2 Vegetable and Fruit Improvement Center, Department of Horticultural Sciences, Molecular and Environmental Plant Sciences Program, MS2133 Texas A&M University, College Station, TX 77843-2133 USA; 3 Institute of Tea, Guizhou Academy of Agricultural Sciences, Guiyang, 550006 Guizhou Province People’s Republic of China; 4 Institute of Agro-bioengineering/College of Life Science, Guizhou University, Huaxi Avenue, Guiyang, 550025 Guizhou Province People’s Republic of China; 5 Key Laboratory of Plant Resources Conservation and Germplasm Innovation in Mountainous Region (Ministry of Education), Guizhou University, Xiahui Road, Huaxi, Guiyang, 550025 Guizhou Province People’s Republic of China

**Keywords:** Genotyping-by-sequencing, Population structure, Linkage disequilibrium, Genetic diversity, Tea plant, Origin center, Guizhou plateau

## Abstract

**Background:**

To efficiently protect and exploit germplasm resources for marker development and breeding purposes, we must accurately depict the features of the tea populations. This study focuses on the *Camellia sinensis* (*C. sinensis*) population and aims to (i) identify single nucleotide polymorphisms (SNPs) on the genome level, (ii) investigate the genetic diversity and population structure, and (iii) characterize the linkage disequilibrium (LD) pattern to facilitate next genome-wide association mapping and marker-assisted selection.

**Results:**

We collected 415 tea accessions from the Origin Center and analyzed the genetic diversity, population structure and LD pattern using the genotyping-by-sequencing (GBS) approach. A total of 79,016 high-quality SNPs were identified; the polymorphism information content (PIC) and genetic diversity (GD) based on these SNPs showed a higher level of genetic diversity in cultivated type than in wild type. The 415 accessions were clustered into three groups by STRUCTURE software and confirmed using principal component analyses (PCA)—wild type, cultivated type, and admixed wild type. However, unweighted pair group method with arithmetic mean (UPGMA) trees indicated the accessions should be grouped into more clusters. Further analyses identified four groups, the Pure Wild Type, Admixed Wild Type, ancient landraces and modern landraces using STRUCTURE, and the results were confirmed by PCA and UPGMA tree method. A higher level of genetic diversity was detected in ancient landraces and Admixed Wild Type than that in the Pure Wild Type and modern landraces. The highest differentiation was between the Pure Wild Type and modern landraces. A relatively fast LD decay with a short range (kb) was observed, and the LD decays of four inferred populations were different.

**Conclusions:**

This study is, to our knowledge, the first population genetic analysis of tea germplasm from the Origin Center, Guizhou Plateau, using GBS. The LD pattern, population structure and genetic differentiation of the tea population revealed by our study will benefit further genetic studies, germplasm protection, and breeding.

**Electronic supplementary material:**

The online version of this article (10.1186/s12870-019-1917-5) contains supplementary material, which is available to authorized users.

## Background

Tea is one of the most popular beverages worldwide [1, 2] with high nutritional and medicinal values. The rich flavor of tea is contributed by nearly 700 bioactive compounds such as catechins (a subgroup of flavan-3-ols), theanine, caffeine, and volatiles [3, 4]. Tea, *Camellia sinensis* (L.) O. Kuntze, Theaceae (*C. sinensis*), has been grown in the Yunnan-Guizhou Plateau in southwest China for approximately 5,000 years and is now widely cultivated all over the world [4]. The Guizhou Plateau is the center of origin of tea [4, 5], where population diversity of the tea is well preserved with abundant wild tea plants, ancient landraces and modern landraces with different morphological characteristics—owing to the unique geology, diverse climates and plentiful rainfall in the region and the cross-pollination nature of tea plants [6]. Large spatial elimination of various tea species has not occurred due to the slow economic development and land use in the Guizhou Plateau.

Ancient tea plants belong to *Sect. Thea* (L.) Dyer, and are defined as varieties grown for more than 100 years. Wild teas, including wild type and self-wild type, are valuable for scientific research and application as they have mainly undergone natural selection and were only minimally affected by artificial selection. Analyzing genetic diversity and population genetic structure is significant to depicte the domestication event and genetic relationships of tea plants. It is also helpful for expediting the development on breeding strategies [7]. Molecular markers have been a powerful tool for the genetic study of tea populations, these include the RAPD [8], nSSR [1, 9], gSSRs [2], SSR [10, 11], SNP [12], AFLP [13], ISSR [14], EST-SSR markers [15, 16], etc. As revealed by these studies, current tea populations evolved from a single species in the Yunnan-Guizhou (Yun-Gui) Plateau. However, the tea populations used in these previous studies had either small sample size or narrow geographic distribution-including only 14 tea-producing regions in Yunan [17], Guangxi [18] or across China.

LD is defined as the association of allelesat different loci within a given population. Understanding the LD pattern is crucial for tea breeding [19–21]. GBS has emerged as a useful tool for linkage map construction and the extensive identification of polymorphisms [21, 23–28]. It has also been widely used in population structure and genetic diversity studies [29–33]. To our knowledge, the LD pattern, population structure, and genetic diversity of tea germplasm had never been examined within previous study using GBS. In addition, very few studies have focused on the tea population in the Guizhou Plateau [22]. Therefore, we employed the GBS approach and performed a genetic analysis on a large tea population consisting of 415 accessions including the wild varieties, ancient landraces and modern landraces in the Guizhou Plateau, as well as cultivated varieties from Zhejiang, Fujian, Hunan, and Guizhou. We aim to (1) identify SNPs at the genome level; (2) analyze the population structure and genetic diversity; and (3) characterize the LD patterns in different varieties. Our findings will facilitate future genome-wide association mapping and marker-assisted selecting of tea.

## Results

### Genome-wide SNPs discovery and the GBS analysis

GBS was performed on 415 tea accessions using Illumina HiSeq X ten. After the primary quality filtering step, 390.3 Gb clean data were obtained with an average of 0.94 Gb clean data per accession (Additional file 1: Table S1). Anaverage of 65% of the total reads were successfully mapped onto the tea genome (Additional file 1: Table S1). The SNPs were detected and genotyped by GATK (version 3.7.0) based on the reference genome [34]. We identified a total of 1,001,372 SNPs with a minimal set of initial quality filters. By restricting the filter conditions, the number of SNPs was subsequently reduced to 287,408, with an average SNP density of one per 10.5 kb and an average quality value of 41,262 (data not shown). The average individual heterozygosity was 17.84% (Additional file 1: Table S2). Furthermore, 79,016 high-quality SNPs were identified and an average individual heterozygosity of 19.21% was observed (Additional file 1: Table S3). All 79,016 SNPs were physically mapped across all scaffolds, with an average map density of 38.24 kb and average quality value of 41,394 (Additional file 1: Table S3). We found more transitions (62,962 loci, 79.68%) than transversions (15,650 loci, 19.81%), and the ratio of transition/transversion was 4.02. C/T transitions and C/G transversions occurred at the highest and lowest frequencies, respectively. The frequencies of A/G and C/T transitions were similar-39.83 and 39.85%, respectively, and the four different types of transversions also occurred at a similar frequency-5.89% for A/T, 5.01% for A/C, 3.81% for G/C and 5.09% for G/T (Table 1).

**Table 1:** Percentage of transition and transversion SNPs identified using genotyping-by-sequencing

	Transitions		Transversions			
	AG	CT	AT	AC	CG	GT
Numbers of allelic sites	31472	31490	4656	3960	3010	4024
Percentage of allelic sites	39.83%	39.85%	5.89%	5.01%	3.81%	5.09%
Total (Percentage)	62962(79.68%)			15650(19.81%)		

### Estimation of genetic diversity

The average genetic diversity (GD), observed heterozygosity (Ho) and polymorphism information content (PIC) of 415 tea accessions were 0.257, 0.247 and 0.214, respectively (Table 2). The percentage of polymorphic loci (PPL) was significantly higher in the cultivation type than in the wild type (Table 2; Additional file 1: Table S5). PPL was significantly higher in the Pure Cultivation Type (GP03) than in the Admixed Wild Type (GP02) and Pure Wild Type (GP01) (Table 3). Among the six zone, PPL was significantly higher in Ia than in Ic, II and III (Additional file 5). GD, Ho, and PIC were significantly higher in the cultivation type than in the wild type (Table 2; Additional file 1: Table S5). GD, Ho, and PIC were significantly higher in the Pure Cultivar Type (GP03) than in the Admixed Wild Type (GP02) and Pure Wild Type (GP01). GD, Ho, and PIC showed significantly higher diversity in Ia, Ib, Ic and II than in III and IV (Table 2; Additional file 1: Table S5; Additional file 5).

**Table 2:** Genetic diversity parameters of 415 tea accessions in Guizhou Plateau

	Group	Number of tested tea accessions			PPL	GD	Ho	PIC
		Cultivation Type	Wild Type	Total				
Region	Ia	106	62	168	0.302(0.007)	0.262(0.003)	0.273(0.004)	0.218(0.002)
	Ib	42	9	51	0.286(0.009)	0.249(0.004)	0.235(0.004)	0.207(0.002)
	Ic	19	38	57	0.258(0.009)	0.239(0.003)	0.231(0.004)	0.199(0.003)
	II	57	26	83	0.276(0.007)	0.250(0.003)	0.237(0.004)	0.208(0.002)
	III	19	22	41	0.267(0.010)	0.230(0.004)	0.209(0.004)	0.192(0.002)
	IV	7	3	10	0.291(0.013)	0.230(0.004)	0.222(0.004)	0.188(0.003)
Growth habits	Cultivation Type	–	–	255	0.298(0.004)	0.253(0.003)	0.259(0.004)	0.210(0.002)
	Wild Type	–	–	160	0.264(0.006)	0.225(0.003)	0.229(0.004)	0.190(0.002)
	Total	–	–	415	2.000(0.000)	0.257(0.003)	0.247(0.004)	0.214(0.002)

*PPL* The percentage of polymorphic loci, *GD* Genetic diversity, *Ho* Observed heterozygosity, *PIC* Polymorphism information content, Pure Wild Type, Admixed Wild Type and Pure Cultivation Type were groups based on STRUCTURE at K = 2 using 415 tea accessions

*Ia* Area with a good suitable climate for tea plant growth in North, Guizhou, *Ib* Area with a good suitable climate for tea plant growth in East, Guizhou, *Ic* Area with a good suitable climate for tea plant growth in South, Guizhou; II, Area with a suitable climate for tea plant growth in center, Guizhou; III, Area with a minor suitable climate for tea plant growth in West, Guizhou; IV, Area with an unsuitable climate for tea plant growth in West, Guizhou

**Table 3:** Genetic differentiation of inferred populations of tea plants in Guizhou Plateau

Group	S	PLL	GD	Ho	PIC	Fis
GP01	52	0.213b	0.129c	0.128c	0.106c	0.017(*p = 0.000*)
GP02	100	0.286a	0.248a	0.276a	0.208a	−0.107(*p = 0.000*)
GP03	263	0.298a	0.254a	0.259b	0.210a	−0.446(*p = 0.000*)
GP03–1	198	0.298a	0.253a	0.260b	0.209a	−0.026(*p = 0.000*)
GP03–2	65	0.297a	0.236b	0.256b	0.194b	−0.078(*p = 0.001*)

*S* Sample size, *PLL* The percentage of polymorphic loci, *GD* Genetic diversity, *Ho* Observed heterozygosity, *PIC* Polymorphism information content, *Fis* Inbreeding coefficient, *p-value*, the statistical significance of the Fis is compared to zero. The different letters indicate a significant difference in a column at *p = 0.05* levels by T-test

### Population structure analysis

We used STRUCTURE and PCA to analyze the genetic structure of the tea accessions. Both analyses were performed using 1,135 LD-pruned SNPs. Based on the genetic distance matrix of the 415 tea accessions, we used TASSEL v.5.2.37 to build an UPGMA tree.

**Fig. 1:**
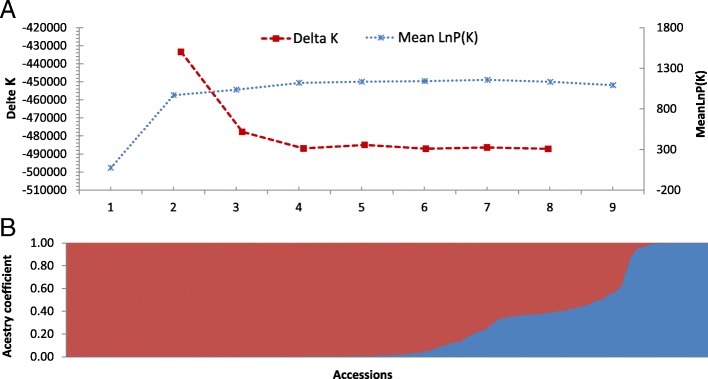
The genetic clusters inferred using STRUCTURE. **a** Graphical method allowing the detection of the number of groups K using ∆K and LnP(K). ∆K and LnP(K) are shown in blue and red, respectively. **b** Inferred population structure of the collection using STRUCTURE software. Bar plot of individual ancestry proportions for the genetic clusters inferred using STRUCTURE (K = 2). Individual ancestry proportions (q values) are sorted within each cluster. Admixture model, independent frequencies, 30,000 burn-in iterations, and 100,000 Markov Chain Monte Carlo iterations were used for this analysis. Cultivation type and wild type ancestral populations are shown in red and blue, respectively

The number of clusters was estimated based on the ΔK method [35, 36] and the plateau criterion [37] in STRUCTURE, firstly. The results showed that the ΔK had the maximum value at K = 2 (Fig. 1a). Based on of this, two ancestral groups were identified (Fig. 1b). Accessions with the score higher than 0.80 were assigned to a pure group, while those with the lower than 0.80 were assigned to the admixture group. The first pure group (referred to as the ‘Pure Wild Type’ or ‘GP01’ from now on) consisted of 52 accessions, all were wild type from *Camellia tachangensis* F.C.Zhang, of which most were from the zones IV, III and II (Additional file 2). One hundred accessions (approximately 24% of 415 populations) exhibited an admixed ancestry. In the admixed cluster (referred to as ‘Admixed Wild Type or GP02’ from now on), 95% were wild type, including 45 *Camellia Tachangensis* from Ia, 50 *Camellia remotiserrata* Zhang from Ia, and five uncertain species (Additional file 2). The second pure group (referred to as the ‘Pure Cultivation Type or GP03’ from now on) consisted of 263 accessions, of which 98% are Cultivated type from *Camellia sinensis* (including the ancient landraces and modern landraces).

**Fig. 2:**
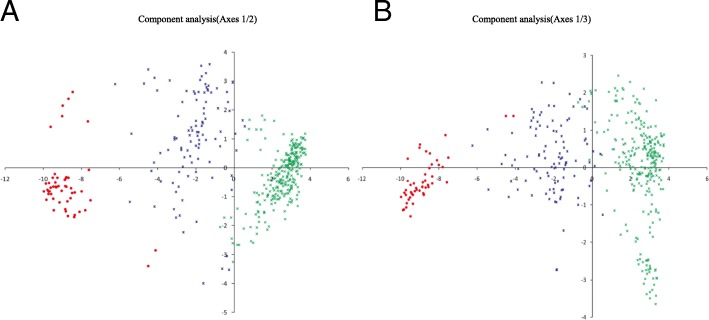
Principal component analysis (PCA) of 415 tea accessions. PCA using 1135 selected SNPs with no linkage disequilibrium in the set of 415 tea accessions. GP03 identified in STRUCTURE is shown in green, GP01 in red and GP02 in blue. First and second components (**a**) and first and third components (**b**) of the PCA analyses are shown

**Fig. 3:**
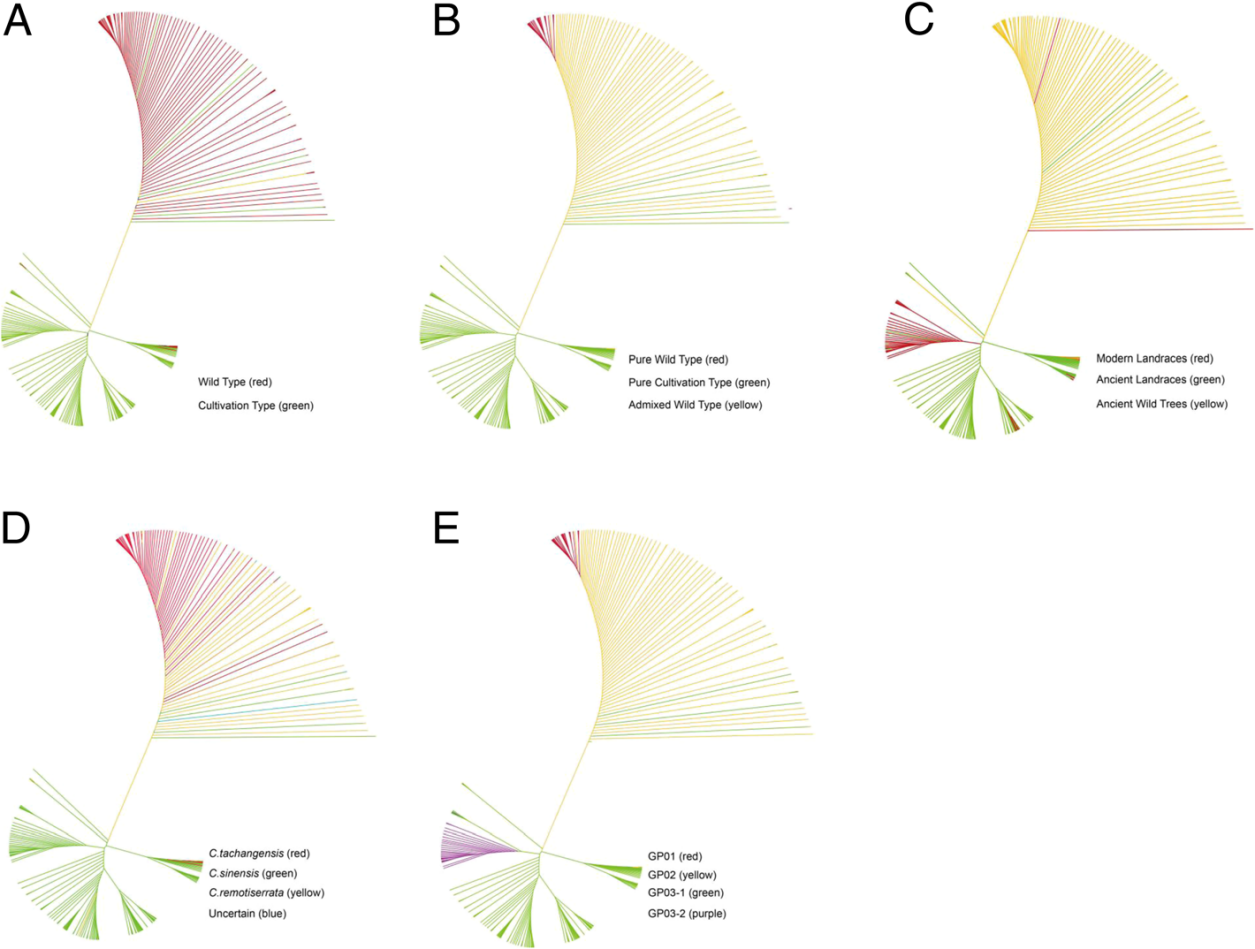
Cluster analysis based on genetic distance using an UPGMA tree. **a** UPGMA cluster tree compared with both growth habits, wild type (red) and cultivation type (green). **b** UPGMA cluster tree compared with STRUCTUER results (k = 2), Pure Wild Type (red), Pure Cultivation Type (green) and Admixed Wild Type (yellow). **c** UPGMA cluster tree compared with growthway, modern cultivation (red), ancient cultivation (green) and wild (yellow). **d** UPGMA cluster tree compared with classification results, *C.tachangensis* (red), *C.sinensis* (green), *C.remotiserrata* (yellow) and uncertain species (blue). **e** UPGMA cluster tree include 4 inferred groups, GP01 (red), GP02 (yellow), GP03–1 (green) and GP03–2 (purple)

The results of PCA analysis were highly consistent with those of STRUCTURE (Fig. 2). PCA revealed two main clusters that correspond to the two ancestral groups identified using STRUCTURE. The Pure Cultivation Type cluster was more scattered than the Pure Wild Type cluster, and the Admixed Wild Type was dispersed between these two clusters along the left side of the PC2 or PC3 axis (Fig. 2). The UPGMA tree also agreed with the STRUCTURE analysis results, although some subgroups were formed in the Pure Cultivation Type clusters (K = 2) (Fig. 3b). Furthermore, the results of UPGMA tree were almost concordant with the growth habits (wild type and cultivation type) (Fig. 3a), the cultivation status (modern landraces, ancient landraces and wild tea trees) (Fig. 3c) and the classification (*C.tachangensis*, *C.sinensis* and *C. remotiserrata*) (Fig. 3d) of tea accessions.

**Fig. 4:**
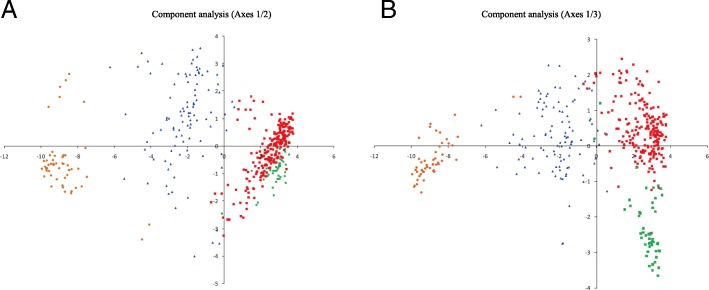
Principal component analysis (PCA) of 415 tea accessions. PCA using 1135 selected SNPs with no linkage disequilibrium in the set of 415 tea accessions. The GP01 cluster identified in STRUCTURE is shown in red, The GP02 cluster in blue, GP03–1 in purple and GP03–2 in green. First and second components (**a**) and first and third components (**b**) of the PCA analyses are shown

The plateau criterion was also used to estimate the number of clusters [37–40]. As shown in Fig. 1, the mean log-likelihood (LnP(K)) curve attained a stable value at around K = 3 ~ 4 [20]. Therefore, we further analyzed the 263 accessions of the GP03 ancestral group to explore whether subgroups could be identified using STRUCTURE reported by Campoy et al. [20]. The 52 accessions in the GP01 ancestral cluster and the 100 accessions in the GP02 cluster were excluded from further analyses (Additional file 2). Within the GP03 group of the 263 accessions, we identified two subgroups at K = 2 (Additional file 3: Figure S1 and S2) based on the Evanno’s ΔK (accessions were assigned into two groups with estimated score of 0.5). The first subgroup included 213 Pure Cultivation Type accessions, of which 78% were ancient landraces (referred to as the ‘ancient landraces’ or ‘GP03–1’ hereafter).The second subgroup was smaller, containing only 50 Pure Cultivation Type accessions, of which 92% were modern landraces (referred to as ‘modern landraces’ or ‘GP03–2’ hereafter) and 8% were breeding varieties (Additional file 2). Overall, the 415 accessions were clustered into three groups, including two main groups (GP01 and GP03) and an admixed group (GP02), and the GP03 group could be further divided into two subgroups (GP03–1 and GP03–2). The result was confirmed by both the UPGMA tree (Fig. 3e) and PCA (Fig. 4) (Additional file 3: Figure S3).

### LD analysis

**Fig. 5:**
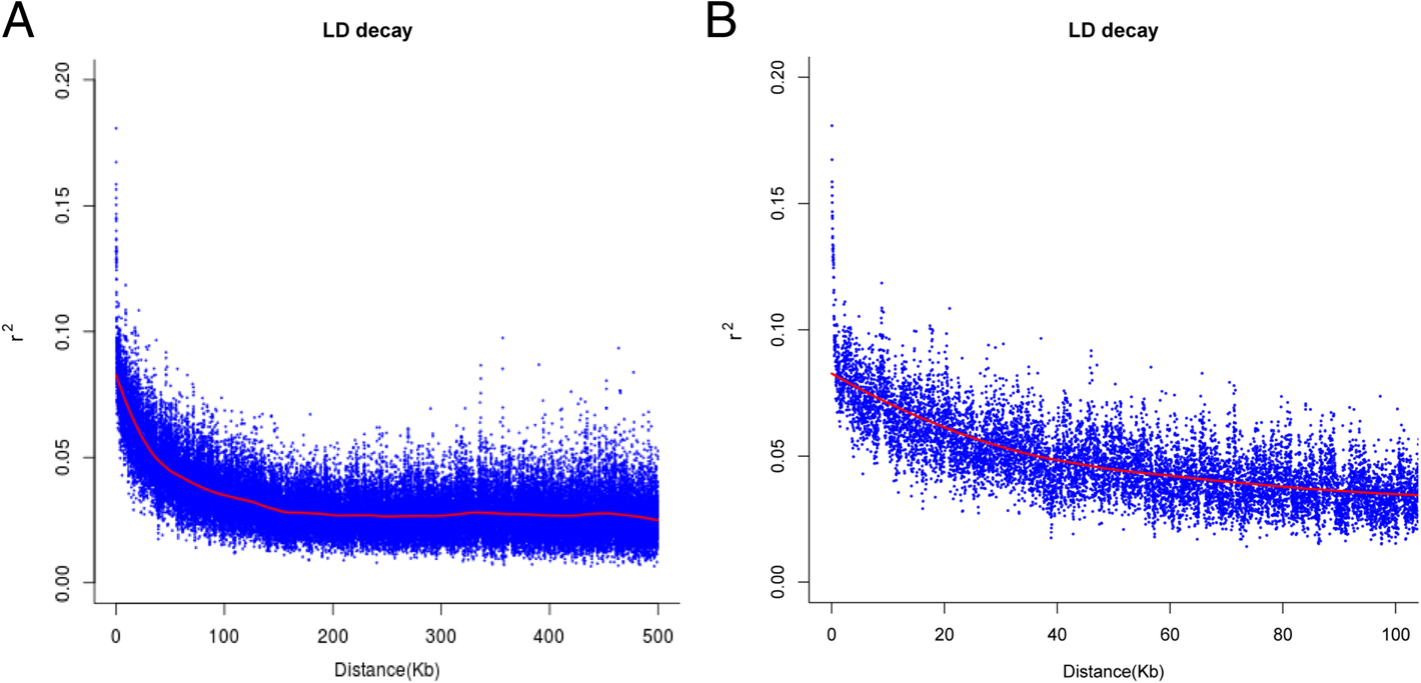
Linkage disequilibrium decay for all scaffolds longer than 500 kb. **a** Scatter plot of LD decay (r^2^) against the genetic distance for pairs of linked SNP across all scaffolds longer than 500 kb. **b** Zoom-in scatter plot of LD decay (r^2^) against the genetic distance

In this study, the extent of LD with a physical distance larger than 500 kb for all scaffolds was evaluated in the 415 tea accessions using 143,041 non-LD-pruned SNPs (Fig. 5a). LD declined rapidly with increasing physical distance. The studied population had an overall low LD and most r^2^ values were below 0.16 (Fig. 5a). On average, LD declined rapidly with an r^2^ value below 0.08 within approximately 2 kb (Fig. 5b).

LD decay in the four inferred groups was estimated (Additional file 4: Figure S1). The lowest LD decay was observed in GP01, as r^2^ reached 0.08 (the threshold) at approximately 35 kb. Conversely, LD declined the most rapidly in GP02—r^2^ = 0.08 corresponded to a physical distance of approximately 1 kb—followed by subgroup GP03–1, in which r^2^ = 0.08 corresponded to approximately 2 kb. The LD of subgroup GP03–2 declined below r^2^ = 0.08 at approximately 25 kb.

### Genetic differentiation analysis

Genetic variation was calculated for the four inferred groups (Table 3). The percentage of polymorphic loci (PPL) was significantly lower in GP01 than in GP02, GP03–1 and GP03–2 (Table 3). We detected no significant differences in PPL among GP02, GP03–1, and GP03–2. The genetic variations in GP02 and GP03–1 were significantly higher than in GP01 and GP03–2, with GP01 showing the lowest genetic variation (Table 3). Fis in all four inferred populations was significantly different than zero (Table 3)-Fis in GP02, GP03–1 and GP03–2 was significantly lower than zero and Fis in GP01 was significantly higher than zero.

The pairwise Fst values ranged from 0.054 to 0.178 with a mean value of 0.101 (Table 4). The lowest level of differentiation was observed between GP03–1 and GP03–2, whereas GP01 and GP03–2 differentiated the most. An intermediate differentiation was observed between GP01 and GP03–1 (Table 4). The Fst results were confirmed by the pairwise genetic distance calculated in the R package adegenet (Table 4).

**Table 4:** Fst and pairwise genetic distance among four inferred populations of tea plant in Guizhou Plateau

Group	GP01	GP02	GP03–1	GP03–2
GP01	–	0.408	0.576	0.599
GP02	0.077c	–	0.253	0.299
GP03–1	0.155b	0.064d	–	0.173
GP03–2	0.178a	0.077c	0.054e	–

The bottom left is the value of Fst; The upper right is the value of pairwise genetic distance; The different letters indicate a significant difference in *p = 0.05* levels by the T-test

## Discussion

### Estimation of genetic diversity

In this study, we report the first genetic diversity analysis of a tea population using GBS-a simple and cost-effective approach [41–44]. We generated 390.30 Gb clean reads and identified 79,016 high-quality SNPs using stringent filtering criteria. The number of SNPs identified in the present study was higher than those used for previous studies [38, 39, 45, 46], suggesting that the GBS approach is powerful for the genetic diversity analyses of tea species.

Previous studies have shown that breeding practices have a greater effect on reducing genetic diversity than domestication, leading to a lower level of genetic diversity in cultivated germplasm compared with wild varieties [7]. Interestingly, our genetic diversity analysis with the Guizhou Plateau tea varieties shows the opposite—we observed a significantly higher genetic diversity level in the cultivation type than in the wild type, which is different from those reported in the previous studies [40, 41]. A plausible explanation for these counterintuitive findings could be due to the existence of ancient landraces in the cultivation type. The ancient landraces were derived from early landraces and their natural offspring, they grow on the edge of terraced fields to prevent soil erosion or used as fences to separate the fields owned by different farmers; such human activities were not for breeding purposes. The cross-pollination characteristics of tea species had also contributed to the large genetic variation in the cultivation type. The relatively isolated natural environment of the Guizhou Plateau may have reduced the genetic perturbations in the wild type group from other tea varieties. Consistent with our hypothesis, a narrow genetic diversity of tea cultivars has been reported in tea-producing regions worldwide where several tea clone cultivars dominated the local populations [32, 33].This will not only impose limitations on tea breeding but also increase the risk of natural hazards because wild tea plants and landraces provide valuable genetic resources for tea-breeding [40]. Such a scenario is especially true for the Guizhou Plateau, which has many ancient landraces and Pure Wild Type accessions, both can be used for tea breeding. Therefore, future studies should focus more on the tea germplasm in the Guizhou Plateau.

### Population structure

In this study, we used three different approaches (STRUCTURE, PCA, and UPGMA) to analyze the population structure of the 415 tea varieties, and the results we obtained complemented the previous studies. STRUCTURE could effectively identify global clusters, which were subsequently validated by PCA. However, the two parameters we used to determine the number of clusters in STRUCTURE yielded different K values—the Evanno’s ΔK method identified K = 2 when analyzing the entire germplasm collection and the cryptic structure. Evanno’s method focuses exclusively on the change in slope, therefore, it estimates the uppermost level structure of the data which may cause ΔK to be artificially maximal at K = 2 in some cases, as reported previously by Campoy JA et al. [20]. We used the maximum likelihood parameter in our analyses as recommended by Pritchard [37], in which K was set to three. K = 3 appeared to fit the origin and the pedigree of the accessions in the Guizhou Plateau. Therefore, the 263 accessions in GP03 obtained with STRUCTURE at K = 2 were further analyzed. The clustering of the tea accessions correlated well with cultivation status origin at K = 2 as revealed by the Evanno’s ΔK method—the 415 accessions were clustered into four populations, including two main populations (GP01 and GP02) and two subgroups (GP03–1 and GP03–2). All accessions in GP01, the Wild Type group, were *C. tachangensis*; the Admixed Wild Type group GP02 contained *C. tachangensis* and *C. remotiserrata* varieties; GP03–1 represented ancient landraces, all of which are *C. sinensis*; and GP03–2 consisted of cultivated varieties including modern landraces and breeding varieties, most of which are *C. sinensis*.

We detected the lowest genetic differentiation and genetic distance between the modern and ancient landraces. The Pure Wild Type and modern landraces exhibited the largest genetic differentiation and genetic distance, followed by that between the Pure Wild Type and ancient landraces, and that between the Admixed Wild Type and ancient landraces. These results support the notion that the evolution of tea plants was related to the historical tea cultivation in the Guizhou Plateau. The Pure Wild Type is the most primitive resource that originated in the region, and the retained species purity was owing to the isolated ecological environment. The ancient landraces and the Admixed Wild Type likely emerged in the Ming Dynasty, when local landraces, introduced landraces, and wild species were co-cultivated. The co-cultivation facilitated cross-pollination among different germplasms, which reduced the genetic distance and differentiation between the ancient landraces and the Admixed Wild Type and significantly increased the diversity of the ancient landraces and the Admixed Wild Type among all inferred groups. Most modern landraces and breeding varieties were assigned to GP03–2, reflecting a narrowed genetic basis of the modern landraces due to breeding practice.

We observed the lowest genetic differentiation between GP03–1 and GP03–2, suggesting that human activities may have caused frequent gene exchange between these two subgroups. GP01 and GP03–2 showed the highest level of genetic differentiation and distance, implying that geographic isolation has restricted the gene flow among populations. This observation could also be a result of the reproductive isolation between species. According to our results, GP03–1 and GP02 exhibited a higher genetic diversity compared with GP01 and GP03–2, therefore, varieties in GP03–1 and GP02 can be used for tea improvement. As revealed by our data, the differences between species did not affect clustering, which reflected the complexity and uncertainty of the tea classification systems. Thus, it is necessary to establish a more scientific classification system. In addition, natural hybridization between tea species may be another explanation of the results mentioned above (Additional file 1: Table S6; Additional file 1: Table S7).

### Linkage disequilibrium

LD decays more rapidly among cross-pollinated species like tea plants than among self-pollinated species due to the less effective recombination in the latter [49, 50]. We observed a rapid LD decay in the 415 accessions—LD declined below *r*^*2*^ = 0.08 at approximately 2 kb, lower than that observed with *Prunus* [20] and melon [21]. This can be due to the self-incompatibility of tea plant [48]. The rapid LD decay and the high proportion of SNPs in LD suggest that GWAS can be used to inform the breeding of the tea varieties in the Guizhou Plateau. These findings are not consistent with those of Jin et al. [5], which may be caused by the differences in the genetic backgrounds among different varieties within each species. In cross-pollinated species, LD can be affected by extreme genetic drift in domestication and breeding during evolution [20]. Thus, we investigated LD decay among the subgroups to provide valuable genetic information for future studies [21]. Subgroups GP01 and GP03–2 displayed a much slower LD decay than GP02 and GP03–1, which is likely because modern landraces had experienced artificial selection pressure and the Pure Wild Type experienced extreme genetic drift, leading to the fixation of a higher number of LD blocks. The slow LD decay in the Admixed Wild Type group and ancient landraces facilities the identification of markers associated with desirable traits, as a relatively small number of markers could cover the entire genome. The Admixed Wild Type group and ancient landraces are ideal populations that can be directly used for breeding—varieties from the Pure Wild Type group can be crossed with modern landraces to achieve heterosis due to a relatively greater genetic distance between these two groups among all.

## Conclusions

Genome-wide SNPs in various tea varieties from the Origin Center, Guizhou Plateau, were identified in this study using GBS. These SNPs were used to analyze the genetic diversity, population structure, and LD pattern of the 415 tea accessions. Our results showed that the 415 accessions could be clustered into four populations, including two main populations (GP01 and GP02) and two subpopulations (GP03–1 and GP03–2). The ancient landrace group was found to have a more complex genetic structure than the wild and modern landraces. These data will inform the collection, conservation, and application of the tea varieties in the Guizhou Plateau.

## Materials and methods

### Plant materials

A total of 415 samples including 159 wild varieties and 256 cultivated varieties (174 ancient landraces, 77 modern landraces and five breeding varieties) were included in this study (Additional file 5; Additional file 2). According to the classification systems reported by Chen et al. [52] and Min [53], 251 *Camellia sinensis* (L.) O. Ktze, 100 *Camellia tachangensis* (F.C.Zhang), 59 *Camellia remotiserrata* (Zhang) and five near *Camellia taliensis* (W.W.Smith) were identified (Additional file 2). Hereafter, samples from the wild tea trees that are more than 100 years old and their natural offsprings are referred to as “wild type”; samples from cultivated tea varieties of more than 100 years old are referred to as “ancient landraces”, and samples from garden tea landraces are referred to as “modern landraces” (Additional file 2). The “ancient landraces”, “modern landraces” and “breeding varieties” that had undergone artificial selection were all referred to as “cultivation type”.

We collected the samples from different tea growing areas with different climates (Additional file 5). Specifically, a total of 276 samples were collected from tea varieties growing in the areas with very suitable climates in Guizhou, these include 168, 51 and 57 accessions in northern (Ia), eastern (Ib) and southern Guizhou (Ic), respectively. Eighty-three samples were harvested from central Guizhou where the climate is suitable for tea growth (II). Forty-one samples were collected from the areas in western Guizhou with a minor suitable climate (III), and 10 samples were from areas in western Guizhou with an unsuitable climate. One variety was collected from Guizhou. Four varieties were collected from other provinces, these include two from Fujian, one from Zhejiang, and one from Hunan (Additional file 5; Additional file 2) [35]. The samples were planted in the city of Guiyang, China. Fresh leaves harvested from each accession were snap frozen in liquid nitrogen and stored at − 80 °C until use.

### DNA extraction

We used the Plant Genomic DNA Rapid Extraction kit (Biomed Gene Technology) to isolate genomic DNA from the samples. DNA integrity was tested on 1% agarose gel, and DNA purity was tested and quantified using Qubit Fluorometer (Invitrogen).

### Library preparation and sequencing

We used 5 U of SacI and MseI (NEB) and 1 × restriction buffer in a 25 μl reaction to digest 100 ng genomic DNA. After digestion, SacAD and MseAD adaptors were ligated to the digested DNA fragments; 12 samples were pooled in equal volumes and purified using the QIAquick PCR Purification Kit (Qiagen) [47]. We then used the PCR Primer Cocktail and PCR Master Mix to amplify the purified DNA fragments. Amplicons of 500–550 bp (including the 120 bp adaptor) were retrieved through electrophoresis using 2% agarose gel and purified using the QIAquick Gel Extraction Kit (Qiagen) [47]. The Agilent DNA 12,000 kit and 2100 Bioanalyzer system (Agilent) were used to determine the average length of DNA fragments, and the resulting DNA libraries were quantified using real-time PCR with a TaqMan probe and sequenced on the Illumina HiSeq X ten platform with the paired-end 150 (PE150) sequencing strategy. Each library contains 48 samples, and we matched the clean reads individually to the barcodes and remnant restriction sites at both ends [47].

### Sequence alignment and SNP identification

The barcodes were used to de-multiplex the raw DNA reads, and a custom perl script was used to trim the adaptors. Only the reads with quality values > 5 were retained as the clean data, and then aligned to the reference genome (http://www.plantkingdomgdb.com/tea_tree/) [3] using BWA-MEM (version 0.7.10) with parameters ‘*-T 20 -k 30*’ [54]. GATK (VERSION 3.7.0) was used call for SNPs.

The SNPs were filtered according to the methods used by Hussain et al. [23], Chen et al. [19] and Eltaher et al. [28] based on the following criteria: (1) variants must be bi-allelic SNPs; (2) “QUAL < 50.0 || QD < 2.0 || FS > 60.0 || MQ < 40.0 || Mapping Quality Rank Sum < -12.5 || Read Pos Rank Sum < -8.0” was used in variant filtration in GATK (version 3.7.0) to filter the SNPs; (3) SNPs with minor allele frequency (MAF) lower than 0.05 or missing data rate higher than 20% were filtered out by VCFtools (version 0.1.15); (4) The SNPs were pruned with a window of 50 SNPs, a step size of 10 SNPs, and an *r*^*2*^ threshold of 0.2 by Plink (v1.9). After the filtering, 415 accessions and 79,016 SNPs were retained and used for further analysis.

### Analysis of genetic diversity

The polymorphism information content (PIC) values for the SNP data were calculated using the following equation [19].

$$ \mathrm{PIC}=1-\sum \limits_{i=1}^n{P}_i^2-\sum \limits_{i=1}^{n-1}\sum \limits_{j=i+1}^n2{P}_i^2{P}_j^2 $$

The mean number of observed alleles per locus and the observed heterozygosity (Ho) were calculated for each group using TASSEL v.5.2.37 [55]. Genetic diversity and inbreeding were calculated for each group using PowerMarker v3.25. Fst was calculated for each group using VCFtools [56].

### Linkage disequilibrium

Prior to the PCA and STRUCTURE analyses, we LD-pruned the SNPs again using Plink (v1.9) [51] with a window of 50 SNPs and a step size of five makers. The *r*^*2*^ threshold was 0.4. PLINK was used to measure pairwise LD between multi-SNPs [20, 54]. The pairwise LD between 143,041 genome-wide unpruned SNPs from sequences longer than 500 kb was calculated based on the allele frequency correlations (*r*^*2*^) using PopLDdecay program1. To summarize the relationship between LD decay, we fitted a locally-weighted linear regression (loess) model to the *r*^*2*^ data [20, 57] using R function ‘loess’ (http://www.R-project.org/) [58] with *r*^*2*^ summarizing both the recombinational and mutational history [59]. The LD decay plot was drawn using R.

### Population structure

Population structure was analyzed using the model-based Bayesian analysis implemented in STRUCTURE [37]. The number of subpopulations (K) was determined using the mean likelihood values in the ΔK method and the lnP (K) values [36, 59] calculated by Structure Harvester [60]. We estimated the variance between replicates by continuously running K = 1–9 to determine the optimal population number [19]. The analysis was conducted with a burn-in of 30,000 iterations followed by 100,000 Markov Chain Monte Carlo (MCMC) replications in three independent runs. No previous information was used to define the clusters. We enforced K to its true value to assess the clustering results. For each given K value, the run with the highest likelihood was used to cluster the accessions. We set the threshold value at 0.8 to distinguish between the pure and mixed groups. PCA was performed using TASSEL v.5.2.37 [55]. We set the threshold value at 0.8 to distinguish between the pure and mixed groups. The genetic distance among different individuals was used for PCA and constructing the UPGMA tree. The UPGMA tree was generated using a simple matching coefficient in TASSEL v.5.2.37 [37]. Fst and pairwise genetic distance among the four inferred groups were calculated in the R package adegenet v.2.1.1 [61].

## Additional files

Additional file 1: **Table S1.** The quality control (QC) data of each sample. **Table S2.** Statistics of individual heterozygosity of 287,408 SNPs based on GBS. **Table S3.**. Statistics of individual heterozygosity of 79,016 SNPs based on GBS. **Table S4.** SNP density of scaffolds based on GBS. **Table S5.** The *p*-value of genetic diversity parameters in Table 2 based on independent-samples T-test. **Table S6.** Genetic diversity parameters of three species of tea plants in Guizhou Plateau. **Table S7.** Fst and pairwise genetic distance among three Species of tea plant in Guizhou Plateau (XLSX 117 kb)

Additional file 2: Information of 415 tea accessions used in this study, including the accession/clone/collection, the accession name, the zone, the cultivation status, growth habits, the species, the STRUCTURE URE-based grouping (Qi ≥0.8) at K = 2, the notes, the source, and the inferred populations (XLSX 45 kb)

Additional file 3: **Figure S1.** Graphical method allowing the detection of the number of groups using ∆K inferred population structure of the 263 Pure Cultivation Type. **Figure S2.** Inferred population structure of the 263Pure Cultivation Type using STRUCTURE software. Bar plot of individual ancestry proportions for the genetic clusters inferred using STRUCTURE (K = 2) and the reduced dataset. Individual ancestry proportions (q values) are sorted within each cluster. Admixture model, independent frequencies, 30,000 burn-in iterations, 100,000 Markov Chain Monte Carlo iterations were used for this analysis. Ancient landraces (GP03–1) and modern landraces (GP03–2) are shown in yellow and green, respectively. **Figure S3.**. Four inferred populations of the 415tea accessions using STRUCTURE (K = 3). GP01 are shown in red, GP02 are shown in red and blue, GP03–1 are shown in blue, and GP03–2 are shown in green. (PDF 207 kb)

Additional file 4: Average LD decay (r^2^) estimated against the genetic distance for pairs of linked SNP across all scaffolds longer than 500 kb in the 415 accessions (ALL) and four inferred groups (GP01, GP02, GP03–1and GP03–2). (PDF 220 kb)

Additional file 5: Geographic distribution of tea accessions analyzed in the current study according to the collection. (A) The geographical position of Guizhou province in China. (B) Agriculture climate regionalization map for tea plant growth in Guizhou Plateau [35]. Ia: Area with a very suitable climate for tea plant growth in North of Guizhou; Ib: Area with a very suitable climate for tea plants growth in East of Guizhou; Ic: Area with a very suitable climate for tea plants growth in South of Guizhou; II: Area with a suitable climate for tea plant growth in Center of Guizhou; III: Area with a minor suitable climate for tea plant growth in West of Guizhou; IV: Area with an unsuitable climate for tea plants growth in West of Guizhou. (PDF 157 kb)

## Data Availability

The plant materials were growing in our resource nursery which are available from the corresponding author on reasonable request. The raw sequence data reported in this study have been deposited in the Genome Sequence Archive [62] in BIG Data Center, Beijing Institute of Genomics (BIG), Chinese Academy of Sciences, under accession number CRA001438 that is publicly accessible at http://bigd.big.ac.cn/gsa. The genotyping of 79,016 SNPs based on GBS in 415 tea accessions have been deposited into the figshare website10.6084/m9.figshare.8343263.

## Abbreviations

Fis: Inbreeding Coefficient
Fst: Fixation Index
GBS: Genotyping-by-sequencing
GD: Genetic diversity
GWAS: Genome-wide association studies
Ho: Observed heterozygosity
LD: Lingkage disequilibrium
PCA: Principal component analyses
PIC: Polymorphism information content
PPL: The percentage of polymorphic loci
UPGMA: Un-weighted pair group method with arithmetic

## References

[1] Wambulwa MC, Meegahakumbura MK, Kamunya S, Muchugi A, Moller M, Liu J (2016). Insights into the genetic relationships and breeding patterns of the African tea germplasm based on nSSR markers and cpDNA sequences. Front Plant Sci.

[2] Liu S, Liu H, Wu A, Hou Y, An Y, Wei C (2017). Construction of fingerprinting for tea plant (*Camellia sinensis*) accessions using new genomic SSR markers. Mol Breeding.

[3] Xia EH, Zhang HB, Sheng J, Li K, Zhang QJ, Kim C (2017). The tea tree genome provides insights into tea flavor and independent evolution of caffeine biosynthesis. Mol Plant.

[4] Wei C, Yang H, Wang S, Zhao J, Liu C, Gao L (2018). Draft genome sequence of *Camellia sinensis* var. sinensis provides insights into the evolution of the tea genome and tea quality. Proc Natl Acad Sci U S A.

[5] Jin JQ, Yao MZ, Ma CL, Ma JQ, Chen L (2016). Association mapping of caffeine content with TCS1 in tea plant and itsrelated specie. Plant physiol bioch.

[6] Niu SZ. Studies on genetic diversity and resistance of wild tea germplasm (Camellia spp.) in Guizhou Province. Doctoral thesis. Guiyang: Guizhou University; 2014.

[7] Chen L, Yang Y, Yu F (2004). Genetic diversity, relationship and molecular discrimination of elite tea germplasms [*Camellia sinensis* (L.),O.Kuntze] revealed by RAPD markers. Mol Plant Breeding.

[8] Kaundun SS, Zhyvoloup A, Park YG (2012). Evaluation of the genetic diversity among elite tea (*Camellia sinensis*varsinensis) genotypes using RAPD markers. Euphytica.

[9] Meegahakumbura MK, Wambulwa MC, Thapa KK, Li MM, Möller M, Xu JC (2016). Indications for Three Independent Domestication Events for the Tea Plant (*Camellia sinensis*(L.) O. Kuntze) and New Insights into the Origin of Tea Germplasm in China and India Revealed by Nuclear Microsatellites. PloS one.

[10] Fang W, Cheng H, Duan Y, Jiang X, Li X (2011). Genetic diversity and relationship of clonal tea (*Camellia sinensis*) cultivars in China as revealed by SSR markers. Plant Syst Evol.

[11] Tan L-Q, Peng M, Xu L-Y, Wang L-Y, Chen S-X, Zou Y (2015). Fingerprinting 128 Chinese clonal tea cultivars using SSR markers provides new insights into their pedigree relationships. Tree Genet Genomes.

[12] Fang W, Meinhardt L, Tan H, Zhou L, Mischke S, Zhang D (2014). Varietal identification of tea (*Camellia sinensis*) using nanofluidic array of single nucleotide polymorphism (SNP) markers. Hortic Res.

[13] Paul S, Wachira FN, Powell W, Waugh R (1997). Diversity and genetic differentiation among populations of Indian and Kenyan tea (*Camellia sinensis* (L.) O. Kuntze) revealed by AFLP markers. Theor Appl Genet.

[14] Yao MZ, Chen L, Liang YR (2008). Genetic diversity among tea cultivars from China, Japan and Kenya revealed by ISSR markers and its implication for parental selection in tea breeding programmes. Plant Breed.

[15] Yao M-Z, Ma C-L, Qiao T-T, Jin J-Q, Chen L (2011). Diversity distribution and population structure of tea germplasms in China revealed by EST-SSR markers. Tree Genet Genomes.

[16] Zhang Y, Zhang X, Chen X, Sun W, Li J (2018). Genetic diversity and structure of tea plant in Qinba area in China by three types of molecular markers. Hereditas.

[17] Zhao D, Yang J, Yang S, Kato K, Luo J (2014). Genetic diversity and domestication origin of tea plant *Camellia taliensis* (*Theaceae*) as revealed by microsatellite markers. BMC Plant Biol.

[18] Jiang C, Zhao W, Zeng Z, Lai X, Wu C, Yuan S (2018). A treasure reservoir of genetic resource of tea plant ( *Camelliasinensis*) in Dayao Mountain. Genet Resour Crop Evol.

[19] Chen W, Hou L, Zhang Z, Pang X, Li Y (2017). Genetic diversity, population structure, and linkage disequilibrium of a Core collection of Ziziphusjujuba assessed with genome-wide SNPs developed by genotyping-by-sequencing and SSR markers. Front Plant Sci.

[20] Campoy JA, Lerigoleurbalsemin E, Christmann H, Beauvieux R, Girollet N, Querogarcía J (2016). Genetic diversity, linkage disequilibrium, population structure and construction of a core collection of *Prunusavium* L. landraces and bred cultivars. BMC Plant Biol.

[21] Pavan S, Marcotrigiano AR, Ciani E, Mazzeo R, Zonno V, Ruggieri V (2017). Genotyping-by-sequencing of a melon ( *Cucumismelo* L.) germplasm collection from a secondary center of diversity highlights patterns of genetic variation and genomic features of different gene pools. BMC Genomics.

[22] Niu SZ, Song QF, Fan WG, Chen ZW (2017). Effects of drought stress on leaf physiological characteristics and root growth of the clone seedlings of wild tea plants. Acta Ecologica Sinica.

[23] Hussain W, Baenziger P, Belamkar V, Guttieri M, Venegas J, Easterly A (2017). Genotyping-by-sequencing derived high-density linkage map and its application to QTL mapping of flag leaf traits in bread wheat. Sci Rep.

[24] Pucher A, Hash C, Wallace J, Han S, Leiser W, Haussmann B (2018). Mapping a male-fertility restoration locus for the a cytoplasmic-genic male-sterility system in pearl millet using a genotyping-by-sequencing-based linkage map. BMC Plant Biol.

[25] Zhang Z, Wei T, Zhong Y, Li X, Huang J (2016). Construction of a high-density genetic map of *Ziziphusjujuba*Mill. Using genotyping by sequencing technology. Tree Genet Genomes.

[26] Ji F, Wei W, Liu Y, Wang G, Zhang Q, Xing Y (2018). Construction of a SNP-based high-density genetic map using genotyping by sequencing (GBS) and QTL analysis of nut traits in Chinese chestnut (*Castaneamollissima* Blume). Front Plant Sci.

[27] Ma GJ, Song QJ, Markell SG, Qi LL (2018). High-throughput genotyping-by-sequencing facilitates molecular tagging of a novel rust resistance gene, R15, in sunflower (*Helianthus annuus* L.). Theor Appl Genet.

[28] Eltaher S, Sallam A, Belamkar V, Emara H, Nower A, Salem K (2018). Genetic diversity and population structure of F Nebraska winter wheat genotypes using genotyping-by-sequencing. Front Genet.

[29] Burrell AM, Pepper AE, Hodnett G, Goolsby JA, Overholt WA, Racelis AE (2015). Exploring origins, invasion history and genetic diversity of *Imperatacylindrica* (L.) P. Beauv. (Cogongrass) in the United States using genotyping by sequencing. Mol Ecol.

[30] Kujur A, Bajaj D, Upadhyaya HD, Das S, Ranjan R, Shree T (2015). Employing genome-wide SNP discovery and genotyping strategy to extrapolate the natural allelic diversity and domestication patterns in chickpea. Front Plant Sci.

[31] Gouesnard B, Negro S, Laffray A, Glaubitz J, Melchinger A, Revilla P (2017). Genotyping-by-sequencing highlights original diversity patterns within a European collection of 1191 maize flint lines, as compared to the maize USDA genebank. TheorAppl Genet.

[32] Schreiber M, Himmelbach A, Börner A, Mascher M. Genetic diversity and relationship of domesticated rye and its wild relatives as revealed through genotyping-by-sequencing. Evol Appl. 2019;12(1):66–77. 10.1111/eva.12624 PMC6304746 30622636

[33] Korinsak Siripar, Tangphatsornruang Sithichoke, Pootakham Wirulda, Wanchana Samart, Plabpla Anucha, Jantasuriyarat Chatchawan, Patarapuwadol Sujin, Vanavichit Apichart, Toojinda Theerayut (2019). Genome-wide association mapping of virulence gene in rice blast fungus Magnaporthe oryzae using a genotyping by sequencing approach. Genomics.

[34] McKenna A, Hanna M, Banks E, Sivachenko A, Cibulskis K, Kernytsky A (2010). The genome analysis toolkit: a MapReduce framework foranalyzing next-generation DNA sequencing data. Genome Res.

[35] Yang SL, Huang ZY. The climatic superiority and regionalization of tea plant in Guizhou. Tillage Cultiv. 1984;(1):2–10 10.13605/j.cnki.52-1065/s.1984.01.001.

[36] Evanno G, Regnaut S, Goudet J (2005). Detecting the number of clusters of individuals using the software STRUCTURE: a simulation study. Mol Ecol.

[37] Pritchard JK, Stephens M, Donnelly P (2000). Inference of population structure using multilocus genotype data. Genetics.

[38] Ravelombola W, Qin J, Shi A, Miller JC, Scheuring DC, Weng Y (2018). Population structure analysis and association mapping for iron deficiency chlorosis in worldwide cowpea (*Vignaunguiculata* (L.) Walp) germplasm. Euphytica.

[39] Pootakham W, Jomchai N, Ruang-Areerate P, Shearman JR, Sonthirod C, Sangsrakru D (2015). Genome-wide SNP discovery and identification of QTL associated with agronomic traits in oil palm using genotyping-by-sequencing (GBS). Genomics.

[40] Yao MZ, Ma CL, Qiao TT, Jin JQ, Chen L (2012). Diversity distribution and population structure of tea germplasms in China revealed by EST-SSR markers. Tree Genet Genomes.

[41] Wachira F, Tanaka J, Takeda Y (2001). Genetic variation and differentiation in tea (*Camellia sinensis*) germplasm revealed by RAPD and AFLP variation. J Hortic Sci and Biotech.

[42] Yang Z, Chen Z, Peng Z, Yu Y, Liao M, Wei S (2017). Development of a high-density linkage map and mapping of the three-pistil gene (Pis1) in wheat using GBS markers. BMC Genomics.

[43] Bhattarai U, Subudhi PK (2018). Identification of drought responsive QTLs during vegetative growth stage of rice using a saturated GBS-based SNP linkage map. Euphytica.

[44] Hackett CA, Milne L, Smith K, Hedley P, Morris J, Simpson CG (2018). Enhancement of Glen Moy x Latham raspberry linkage map using GbS to further understand control of developmental processes leading to fruit ripening. BMC Genet.

[45] Gardner KM, Brown P, Cooke TF, Cann S, Costa F, Bustamante C (2014). Fast and cost-effective genetic mapping in apple using next-generation sequencing. G3-Genes Genom Genet.

[46] Palero F, Lopes J, Abelló P, Macpherson E, Pascual M, Beaumont M (2009). Rapid radiation in spiny lobsters (*Palinurus*spp) as revealed by classic and ABC methods using mtDNA and microsatellite data. BMC Evol Biol.

[47] Elshire RJ, Glaubitz JC, Sun Q, Poland JA, Kawamoto K, Buckler ES, Mitchell SE (2011). A robust, simple genotyping-by-sequencing (GBS) approach for high diversity species. PLoS One.

[48] Gaut B, Long A (2003). The lowdown on linkage disequilibrium. Plant Cell.

[49] Maruki T, Lynch M (2014). Genome-wide estimation of linkage disequilibrium from population-level high-throughput sequencing data. Genetics.

[50] Zhu X, Dong L, Jiang L, Li H, Sun L, Zhang H (2016). Constructing a linkage-linkage disequilibrium map using dominant-segregating markers. DNA Res.

[51] Purcell S, Neale B, Todd-Brown K, Thomas L, Ferreira M, Bender D (2007). PLINK: a tool set for whole-genome association and population-based linkage analyses. Am J Hum Genet.

[52] Chen L, Yu FL, Tong QQ (2000). Discussions on phylogenetic classification and evolution of sect. *Thea*. J Tea Sci.

[53] Min TL (1992). A revision of *Camellia*sect.*thea*. Acta Bot Yunnanica.

[54] Li H. Aligning sequence reads, clone sequences and assembly contigs with BWA-MEM. arXiv Preprint at https://arxiv.org/abs/1303.3997. 2013.

[55] Bradbury PJ, Zhang Z, Kroon DE, Casstevens TM, Ramdoss Y, Buckler ES (2007). TASSEL: software for association mapping of complex traits in diverse samples. Bioinformatics.

[56] Danecek P, Auton A, Abecasis G, Albers CA, Banks E, Depristo MA (2011). The variant call format and VCFtools. Bioinformatics.

[57] Chao S, Dubcovsky J, Dvorak J, Luo MC, Baenziger SP, Matnyazov R (2010). Population and genome-specific patterns of linkage disequilibrium and SNP variation in spring and winter wheat (*Triticumaestivum* L.). BMC Genomics.

[58] Coreteam R (2015). R: a language and environment for statistical computing. Computing.

[59] Flint-Garcia SA, Thornsberry JM, Th BE (2003). Structure of linkage disequilibrium in plants. Annu RevPlant Biol.

[60] Earl DA, Vonholdt BM (2012). Structure harvester: a website and program for visualizing structure output and implementing the Evanno method. Conserv Genet Resour.

[61] Jombart Thibaut, Ahmed Ismaïl (2011). adegenet 1.3-1: new tools for the analysis of genome-wide SNP data. Bioinformatics.

[62] Wang Y, Song F, Zhu J, Zhang S, Yang Y, Chen T, Tang B, Dong L, Ding N, Zhang Q (2017). GSA: Genome sequence archive*. Genom Proteom Bioinf.

