# Supplementary material for: Genetic diversity, linkage disequilibrium, and population structure analysis of the tea plant (Camellia sinensis) from an origin center, Guizhou plateau, using genome-wide SNPs developed by genotyping-by-sequencing
Source: BMC Plant Biol. 2019 Jul 23;19:328. doi: 10.1186/s12870-019-1917-5 (PMC6652003; doi:10.1186/s12870-019-1917-5)
Supplement: Supplementary file 3 — Figure S1. Graphical method allowing the detection of the number of groups using ∆K inferred population structure of the 263 Pure Cultivation Type. Figure S2. Inferred population structure of the 263Pure Cultivation Type using STRUCTURE software. Bar plot of individual ancestry proportions for the genetic clusters inferred using STRUCTURE (K = 2) and the reduced dataset. Individual ancestry proportions (q values) are sorted within each cluster. Admixture model, independent frequencies, 30,000 burn-in iterations, 100,000 Markov Chain Monte Carlo iterations were used for this analysis. Ancient landraces (GP03–1) and modern landraces (GP03–2) are shown in yellow and green, respectively. Figure S3.. Four inferred populations of the 415tea accessions using STRUCTURE (K = 3). GP01 are shown in red, GP02 are shown in red and blue, GP03–1 are shown in blue, and GP03–2 are shown in green. (PDF 207 kb) [file 12870_2019_1917_MOESM3_ESM.pdf]

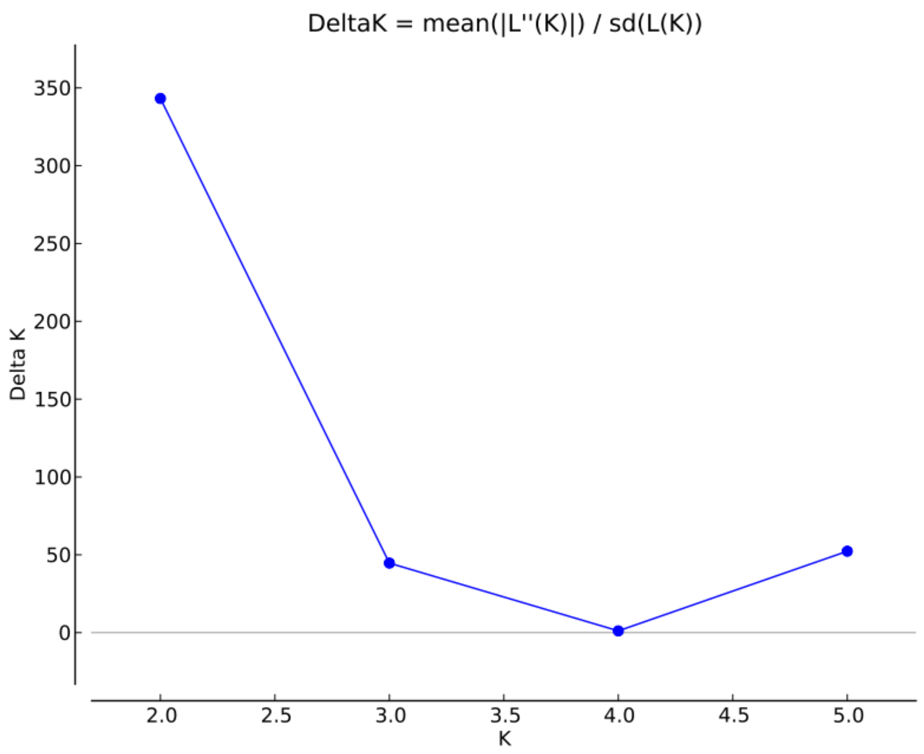

Fig S1 Graphical method allowing the detection of the number of groups using ΔK Inferred population structure of the 263 Pure Cultivation Type

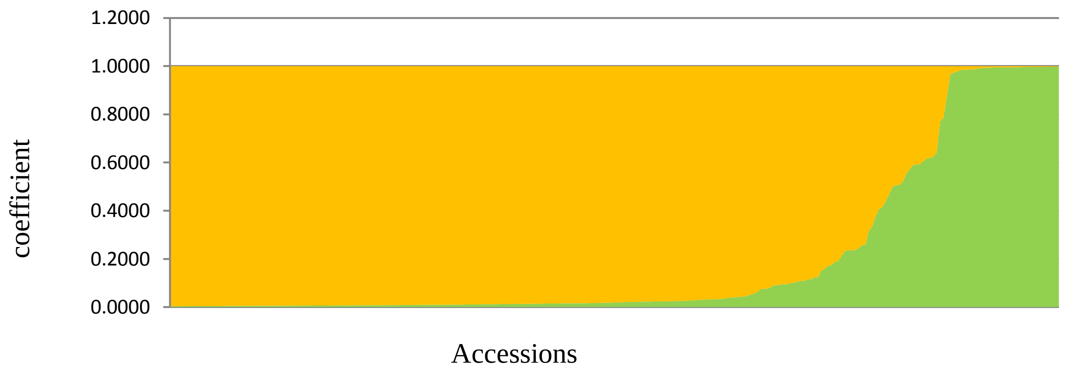

Fig S2 Inferred population structure of the 263 Pure Cultivation Type using STRUCTURE software. Bar plot of individual ancestry proportions for the genetic clusters inferred using STRUCTURE (K = 2) and the reduced dataset. Individual ancestry proportions (q values) are sorted within each cluster. Admixture model, independent frequencies, 30,000 burn-in iterations, 100,000 Markov Chain Monte Carlo iterations were used for this analysis. Ancient landraces (GP03-1) and modern landraces (GP03-2) are shown in yellow and green, respectively

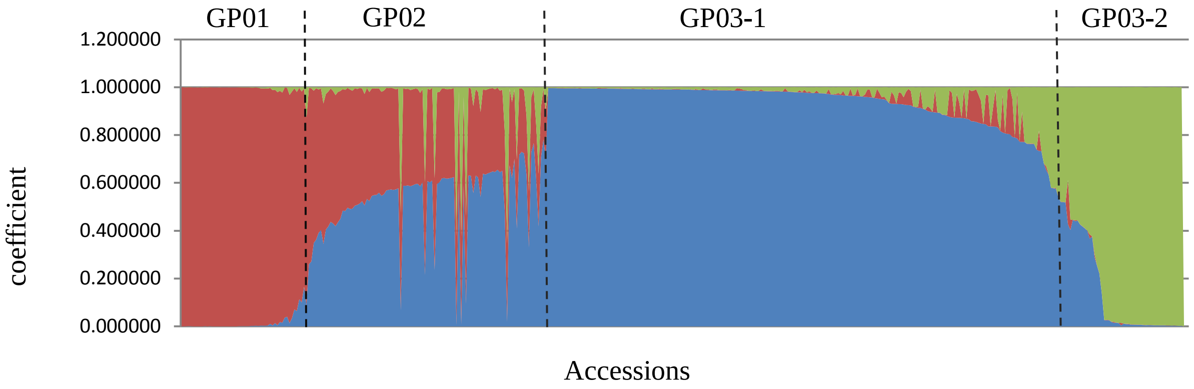

Fig S3 Four inferred population structure of the 415 tea accessions using STRUCTURE (K = 3). GP01 are shown in red, GP02 are shown in red and blue, GP03-1 are shown in blue, GP03-2 are shown in green, respectively.
