# Supplementary figures and images for: Genetic diversity, linkage disequilibrium, and population structure analysis of the tea plant (Camellia sinensis) from an origin center, Guizhou plateau, using genome-wide SNPs developed by genotyping-by-sequencing
Source: BMC Plant Biol. 2019 Jul 23;19:328. doi: 10.1186/s12870-019-1917-5 (PMC6652003; doi:10.1186/s12870-019-1917-5)

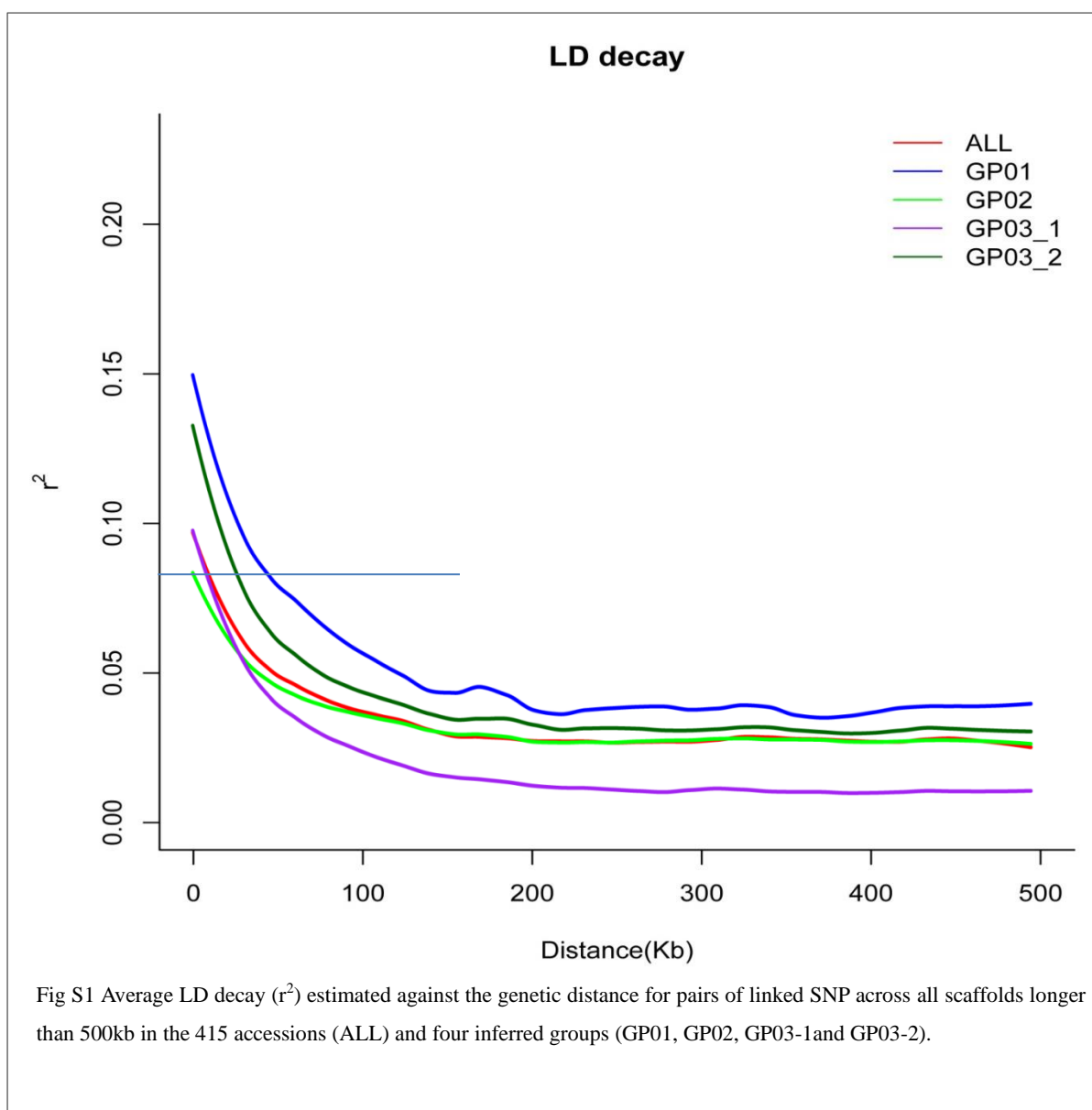

Supplement: Supplementary file 4 — Average LD decay (r2) estimated against the genetic distance for pairs of linked SNP across all scaffolds longer than 500 kb in the 415 accessions (ALL) and four inferred groups (GP01, GP02, GP03–1and GP03–2). (PDF 220 kb) [file 12870_2019_1917_MOESM4_ESM.pdf]
