# Supplementary material for: Genetic diversity, linkage disequilibrium, and population structure analysis of the tea plant (Camellia sinensis) from an origin center, Guizhou plateau, using genome-wide SNPs developed by genotyping-by-sequencing
Source: BMC Plant Biol. 2019 Jul 23;19:328. doi: 10.1186/s12870-019-1917-5 (PMC6652003; doi:10.1186/s12870-019-1917-5)
Supplement: Supplementary file 5 — Geographic distribution of tea accessions analyzed in the current study according to the collection. (A) The geographical position of Guizhou province in China. (B) Agriculture climate regionalization map for tea plant growth in Guizhou Plateau [35]. Ia: Area with a very suitable climate for tea plant growth in North of Guizhou; Ib: Area with a very suitable climate for tea plants growth in East of Guizhou; Ic: Area with a very suitable climate for tea plants growth in South of Guizhou; II: Area with a suitable climate for tea plant growth in Center of Guizhou; III: Area with a minor suitable climate for tea plant growth in West of Guizhou; IV: Area with an unsuitable climate for tea plants growth in West of Guizhou. (PDF 157 kb) [file 12870_2019_1917_MOESM5_ESM.pdf]

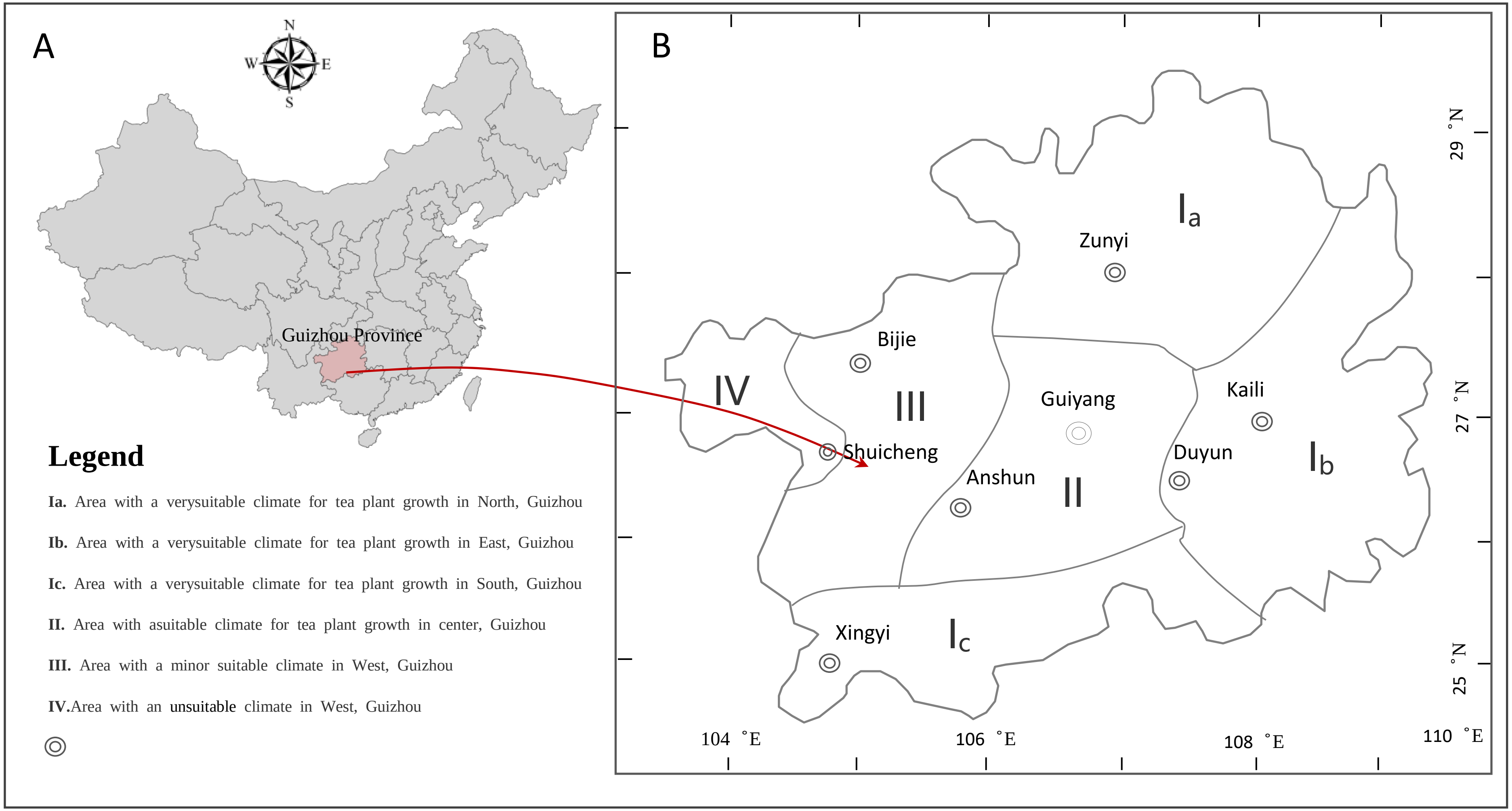
A
N
W
E
S
Guizhou Province
Legend
Ia. Area with a verysuitable climate for tea plant growth in North, Guizhou
Ib. Area with a verysuitable climate for tea plant growth in East, Guizhou
Ic. Area with a verysuitable climate for tea plant growth in South, Guizhou
II. Area with asuitable climate for tea plant growth in center, Guizhou
III. Area with a minor suitable climate in West, Guizhou
IV.Area with an unsuitable climate in West, Guizhou
B
Ia
Zunyi
Bijie
IV
III
Guiyang
Kaili
Shuicheng
Duyun
Ib
Anshun
II
Xingyi
Ic
29 °N
27 °N
25 °N
104 °E
106 °E
108 °E
110 °E
